# Use of Hearing Services in Traditional Medicare and Medicare Advantage

**DOI:** 10.1001/jamahealthforum.2024.3619

**Published:** 2024-10-25

**Authors:** Sarah Y. Bessen, Emmanuel E. Garcia Morales, Frank R. Lin, Nicholas S. Reed

**Affiliations:** 1 Cochlear Center for Hearing and Public Health, Johns Hopkins University, Baltimore, Maryland; 2 Department of Otolaryngology–Head and Neck Surgery, Johns Hopkins University, Baltimore, Maryland; 3 Department of Epidemiology, Johns Hopkins Bloomberg School of Public Health, Baltimore, Maryland; 4 Optimal Aging Institute, NYU Grossman School of Medicine, New York, New York

## Abstract

This cross-sectional study compares use of hearing evaluations and hearing aids among Medicare Advantage and traditional Medicare beneficiaries with hearing loss.

## Introduction

Hearing aids and most related hearing services are statutory exclusions under traditional Medicare (TM), requiring beneficiaries to face high out-of-pocket costs or navigate a complex network of Medicare Advantage (MA) plans for coverage. Nearly 90% of Medicare beneficiaries who report difficulty hearing do not own hearing aids.^1^ Our aim was to compare MA and TM beneficiaries’ use of hearing evaluations and hearing services.

## Methods

This cross-sectional analysis pooled the 2019-2021 cycles of the Medicare Current Beneficiary Survey (MCBS), a nationally representative panel survey of the Medicare population. The MCBS is approved by the NORC at the University of Chicago institutional review board, which waived informed consent due to use of publicly available, deidentified data. The study followed the STROBE reporting guideline.

We grouped adult beneficiaries (aged ≥65 years) into 5 mutually exclusive categories based on presence of additional coverage: TM only, Medicaid, employer sponsored, MA, and Medigap. Use of hearing services (routine hearing examinations, hearing aid fittings, purchase of hearing aids) within the past year was derived from the Hearing Utilization Events survey. These measures are described more fully in eMethods 1 to 3 in Supplement 1). For participants with responses in multiple survey cycles, only the most recent responses were considered.

We estimated percentages using multivariable logistic regression adjusted for demographic (age and self-reported sex and race and ethnicity [Asian, Black, Hispanic, White, other (including American Indian or Alaska Native, Native Hawaiian or Pacific Islander, more than 1 race or ethnicity, or other not otherwise specified)] derived from the MCBS), socioeconomic, and health characteristics (eMethods 4 in Supplement 1) to assess the association between insurance type and hearing services use. A sensitivity analysis was restricted to participants with self-reported hearing loss or hearing aid use. Survey weights were used to account for the complex MCBS design. Analyses were performed between March 28 and July 20, 2024, using Stata, version 18.0 (StataCorp LLC).

## Results

In a sample of 19 818 MCBS participants (mean [SD] age, 77.0 [7.3] years; 55.4% female, 44.6% male), a weighted 48.7% reported hearing loss or hearing aid use (Table 1). Compared with beneficiaries with only TM (10.9%), MA beneficiaries (28.6%) had similar estimated adjusted percentages of hearing service use, including routine hearing tests (TM, 4.2% [95% CI, 3.3%-5.1%]; MA, 5.3% [95% CI, 4.8%-5.9%]), hearing aid fittings (TM, 3.2% [95% CI, 2.4%-4.0%]; MA, 3.6% [95% CI, 3.1%-4.1%]), and hearing aid purchases (TM, 2.1% [95% CI, 1.5%-2.7%]; MA, 2.4% [95% CI, 2.0%-2.9%]) (Table 2). The sensitivity analysis limited to beneficiaries with self-reported hearing loss or hearing aid use had greater use of routine hearing examinations in MA (TM, 7.1% [95% CI, 5.5%-8.7%]; MA, 9.9% [8.9%-10.9%]).

**Table 1.** Baseline Characteristics of Medicare Beneficiaries by Insurance Coverage Type, Medicare Current Beneficiary Survey 2019-2021^a^

Characteristic	No. of participants (%)						*P* value
	Total (N = 19 818)	Traditional Medicare only (n = 2073)	Medicaid (n = 2474)	Employer sponsored (n = 4464)	Medicare Advantage (n = 6266)	Medigap (n = 4541)	
Age, mean (SD), y	77.0 (7.3)	77.2 (7.4)	77.2 (7.8)	76.3 (7)	77.4 (7.2)	77.0 (7.2)	<.001
Sex							
Female	11 007 (55.4)	994 (46.4)	1686 (66.6)	2335 (52.7)	3423 (55.4)	2569 (57.3)	<.001
Male	8811 (44.6)	1079 (53.6)	788 (33.4)	2129 (47.3)	2843 (44.6)	1972 (42.7)	
Race and ethnicity							
Asian	274 (2.3)	23 (1.8)	93 (5.4)	50 (2.2)	77 (2.4)	31 (1)	<.001
Black	1729 (9.7)	184 (10.9)	516 (23)	303 (8.1)	597 (10.6)	129 (3.4)	
Hispanic	699 (2.5)	44 (1.8)	436 (12.7)	40 (1)	158 (1.9)	21 (0.3)	
White	16702 (83.1)	1754 (81.3)	1352 (55.9)	3996 (86.4)	5288 (82.5)	4312 (93.9)	
Other^b^	414 (2.5)	68 (4.2)	77 (3)	75 (2.3)	146 (2.6)	48 (1.4)	
Self-reported health							
Excellent	3729 (19.9)	389 (18.6)	235 (9.3)	946 (23.5)	1227 (19.8)	932 (21.6)	<.001
Very good	6817 (35)	678 (32.9)	469 (19.3)	1690 (38)	2230 (36.1)	1750 (38.9)	
Good	5903 (29.1)	653 (31.5)	854 (34.2)	1272 (27.5)	1871 (29.4)	1253 (26.9)	
Fair	2682 (12.7)	277 (13.6)	708 (28.4)	453 (8.8)	757 (11.8)	487 (10.2)	
Poor	687 (3.3)	76 (3.4)	208 (8.8)	103 (2.3)	181 (2.9)	119 (2.4)	
Education							
Less than high school	2944 (11.7)	302 (12.3)	1175 (40.5)	232 (4.4)	892 (11.9)	343 (5.5)	<.001
High school graduate	9340 (46.5)	1066 (52.8)	982 (45.2)	1941 (41.2)	3180 (50.1)	2171 (45.7)	
Some college or more	7534 (41.8)	705 (34.9)	317 (14.3)	2291 (54.4)	2194 (37.9)	2027 (48.9)	
Living arrangement							
Alone	6449 (30.1)	703 (32.7)	1095 (45.5)	1290 (25.3)	1933 (28.7)	1428 (28.4)	<.001
Spouse only	8559 (45.7)	826 (40.4)	335 (14.9)	2322 (53.7)	2746 (45.8)	2330 (53.8)	
Children or family^c^	3065 (14.3)	367 (16.7)	661 (23.1)	555 (13)	1003 (14.8)	479 (9.9)	
Other^d^	1745 (10)	177 (10.2)	383 (16.6)	297 (8)	584 (10.8)	304 (7.8)	
Metropolitan residence	15 609 (82.3)	1469 (74.7)	1941 (80.7)	3605 (85.1)	5284 (87)	3310 (77.8)	<.001
Income, % of FPL							
<200	8749 (41.7)	901 (42.9)	2382 (95.1)	1048 (23.6)	2863 (44.4)	1555 (32.5)	<.001
200-399	5240 (25.8)	595 (29.7)	82 (4.6)	1243 (24.8)	1932 (30.5)	1388 (29.4)	
≥400	5829 (32.5)	577 (27.4)	10 (0.3)	2173 (51.6)	1471 (25)	1598 (38.1)	
No. of ADL difficulties							
0	14 532 (77.4)	1464 (73.9)	1309 (57.1)	3463 (82.4)	4741 (78.4)	3555 (81.8)	<.001
1	2593 (11.4)	313 (13.4)	425 (16.4)	540 (9.9)	810 (11.9)	505 (9.3)	
≥2	2693 (11.2)	296 (12.6)	740 (26.5)	461 (7.8)	715 (9.8)	481 (8.9)	
Chronic conditions							
Cancer	4061 (18.3)	359 (15.2)	441 (16.4)	969 (18.8)	1244 (18)	1048 (20.5)	<.001
Stroke	2037 (8.8)	224 (9.4)	403 (15.3)	394 (6.6)	609 (8.9)	407 (7.9)	<.001
COPD	3472 (16.5)	365 (16.9)	626 (25.7)	725 (14.5)	1027 (16)	729 (14.7)	<.001
Depression	3971 (20.6)	380 (19.1)	784 (31.2)	805 (18)	1163 (20.2)	839 (19.6)	<.001
Diabetes	6183 (30.6)	650 (31.2)	1040 (39.8)	1284 (28.4)	2005 (32.1)	1204 (26.4)	<.001
Chronic heart failure	1312 (5.4)	161 (6.2)	270 (9.9)	242 (3.7)	374 (5)	265 (5)	<.001
Hip fracture	785 (3.1)	98 (3.9)	127 (4.1)	158 (2.6)	217 (2.8)	185 (3.3)	.001
Hypertension	12 896 (61.7)	1293 (59.5)	1871 (72.4)	2786 (58.3)	4099 (63.3)	2847 (59.2)	<.001
Ischemic heart disease	1769 (7.9)	222 (9.5)	239 (8.6)	428 (7.9)	484 (7.2)	396 (7.7)	<.001
Arthritis	4140 (18.8)	402 (17.5)	867 (32.7)	759 (15.1)	1272 (19)	840 (16.7)	<.001
Functional hearing loss	10 611 (48.7)	1133 (49.4)	1170 (44.2)	2446 (48.8)	3333 (48.9)	2529 (50.3)	<.001

Abbreviations: ADL, activities of daily living; COPD, chronic obstructive pulmonary disease; FPL, federal poverty level.

^a^ Medicare Current Beneficiary Survey analytic weights were applied to all analyses.

^b^ Included American Indian or Alaska Native, Native Hawaiian or Pacific Islander, more than 1 race or ethnicity, or other not otherwise specified on the survey instrument.

^c^ “Family” includes spouse and children, spouse and grandchildren, or spouse and children and grandchildren.

^d^ Includes those who answered partners only or partners and children.

**Table 2.** Hearing Services Use by Insurance Coverage, Medicare Current Beneficiary Survey 2019-2021

Insurance type	Estimated adjusted % (95% CI)^a^		
	Routine hearing examination	Hearing aid fitting	Hearing aid purchase
Traditional Medicare only	4.2 (3.3-5.1)	3.2 (2.4-4.0)	2.1 (1.5-2.7)
Medicare Advantage	5.3 (4.8-5.9)	3.6 (3.1-4.1)	2.4 (2.0-2.9)
Medicaid	4.7 (3.5-5.9)	3.5 (2.4-4.6)	2.0 (1.2-2.9)
Employer	6.3 (5.5-7.0)	4.9 (4.2-5.5)	3.4 (2.8-4.0)
Medigap	5.7 (5.0-6.5)	3.5 (3.0-4.0)	3.1 (2.5-3.6)

^a^ Multivariable logistic regression models were adjusted for age, gender, race and ethnicity, income relative to the federal poverty level, living arrangement, urban residence, self-reported health, number of difficulties with activities of daily living, comorbid chronic conditions (stroke, chronic obstructive pulmonary disease, depression, diabetes, chronic heart failure, hip fracture, hypertension, arthritis, ischemic heart disease), and survey year.

## Discussion

In this cross-sectional study, Medicare beneficiaries with MA plans and TM used hearing services at similar rates overall, but MA beneficiaries with hearing loss or hearing aid use were more likely to receive hearing examinations. Further research is needed to better understand the use of hearing services in MA and other categories of Medicare coverage.

The broader literature shows mixed quality benefits of MA compared with TM, though recent work estimated that Medicare spent 6% more on MA enrollees compared with similar TM enrollees in 2023.^2^ Our findings are consistent with research suggesting similar use of dental and vision services (also excluded from TM) between MA and TM beneficiaries.^3,4^

While nearly all MA enrollees have access to hearing benefits, including 97% in 2021, the observed similar use rates between TM and MA beneficiaries may correlate with widespread variation in generosity or beneficiary awareness of MA plan benefits.^5^ A limitation of our study was the inability to assess heterogeneity of hearing care benefits across MA plans. Further research, including review of individual plan benefits, is needed to understand how specific MA plan features correlate with use of hearing services among Medicare beneficiaries with hearing loss.

## Notes

Supplement 1. **eMethods 1.** Hearing Utilization Events Survey **eMethods 2.** Insurance Coverage Category Definitions **eMethods 3.** Creation of Insurance Coverage Categories **eMethods 4.** Covariates
eReference

Supplement 2.
Data Sharing Statement
