# Supplementary material for: Use of Hearing Services in Traditional Medicare and Medicare Advantage
Source: JAMA Health Forum. 2024 Oct 25;5(10):e243619. doi: 10.1001/jamahealthforum.2024.3619 (PMC11581654; doi:10.1001/jamahealthforum.2024.3619)
Supplement: Supplement 1. — eMethods 1. Hearing Utilization Events Survey eMethods 2. Insurance Coverage Category Definitions eMethods 3. Creation of Insurance Coverage Categories eMethods 4. Covariates eReference [file jamahealthforum-e243619-s001.pdf]

Supplemental Online Content

Bessen SY, Garcia Morales EE, Lin FR, Reed NS. Use of hearing serives in traditional Medicare and Medicare Advantage. *JAMA Health Forum*. 2024;5(10):e243619. doi:10.1001/jamahealthforum.2024.3619

eMethods 1. Hearing Utilization Events Survey

eMethods 2. Insurance Coverage Category Definitions

eMethods 3. Creation of Insurance Coverage Categories

eMethods 4. Covariates

eReference

This supplemental material has been provided by the authors to give readers additional information about their work.

© 2024 Bessen S et al. *JAMA Health Forum.*

eMethods 1. Hearing Utilization Events Survey

The Hearing Utilization Events (HUE) survey segment was added to the Medicare Current Beneficiary Survey (MCBS) in 2019 and includes data on individual hearing care events reported during a participant interview or created from Medicare claims data.

The unit of observation is a single visit to a hearing care provider (e.g. otolaryngologist or audiologist). Multiple services can be rendered during a single hearing event. A beneficiary may be represented multiple times in HUE if they report multiple hearing events. However, a beneficiary will not appear in HUE if they do not have any hearing events to report in the interview or there is no relevant Medicare claims data. While HUE data is derived from both survey-reported and claims data, the majority of data is survey-reported because MCBS claims data is derived from traditional Medicare fee-for-service claims, which does not cover the majority of hearing events.

The following survey questions are used to characterize the hearing events used in the present study.

1. Since (REFERENCE DATE/UTILDATE)/Between (REFERENCE DATE) and (DATE OF DEATH/DATE OF INSTITUTIONALIZATION/ENDUTILD)], did [you/(SP)] go to a doctor or any other person for hearing care? [Hearing care providers include otorhinolaryngologists (ear nose and throat doctors), otologists, neurotologists, audiologists, audiometrists, and hearing instrument specialists.]"

2. For [your/(SP's)] [VISIT ON EVENT DATE], what did [you/(SP)] have done? CHECK ALL THAT APPLY

    a. Hearing aid fitting/evaluation
    b. Hearing aid purchase/repair
    c. Hearing exam
    d. Ear wax removal
    e. Ear ventilation tubes
    f. Tympanoplasty (reconstruction of ear drum)
    g. Cochlear implant surgery
    h. Hearing rehabilitative services
    i. Other
    j. Don't know
    k. Refused

eMethods 2. Insurance Coverage Category Definitions[1]

Traditional Medicare (TM) provides health insurance to adults 65 or older and younger adults with long-term disabilities. For the purposes of this study, only beneficiaries aged 65 years and older were included. TM includes Medicare Part A (Hospital Insurance) and Part B (Medical Insurance).

Medicare Advantage (MA) refers to supplemental private health insurance plans, such as an Health Maintenance Organization or Preferred Provider Organization. These plans include Medicare Part A and Part B, usually include Part D (Prescription drug coverage), and may offer additional benefits that are excluded from Traditional Medicare (e.g. hearing, vision, and dental benefits).

Medicaid is the federal-state program that provides insurance coverage to low-income individuals.

Medigap refers to Medicare supplement insurance policies that are sold by private insurance companies. These policies only serve to fully or partially cover cost-sharing requirements of TM (Medicare Part A and Part B).

Employer-sponsored health insurance refers to health insurance coverage under a plan sponsored by an employer or union. Employer-sponsored health insurance may include benefits for both retirees as well as actively working individuals.

Of note, most Medicare beneficiaries have coverage through Medicare Advantage or Traditional Medicare coupled with other supplemental insurance options, such as Medicaid, Medigap, or Employer-Sponsored coverage.

eMethods 3. Creation of Insurance Coverage Categories

Information on beneficiary insurance coverage was derived from MCBS survey data. Beneficiaries were grouped into five mutually exclusive categories based on presence of additional coverage: Traditional Medicare only, Medicaid, Employer-Sponsored, Medicare Advantage, and Medigap. For the purposes of this study, beneficiaries were assigned using the following insurance categorization system. The system first categorizes individuals as having Traditional Medicare, due to inclusion criteria of MCBS. Next, participants are assigned into the Medicaid category, regardless of their other insurance statuses. After Medicaid is assigned, the next category assigned is Employer-Sponsored insurance, followed by Medicare Advantage and Medigap. If a participant does not have Medicaid, Employer-Sponsored insurance, Medicare Advantage, or Medigap, they remain as having Traditional Medicare Only. Overall, the insurance hierarchy is as follows: Medicaid > Employer-Sponsored > Medicare Advantage > Medigap > Traditional Medicare Only.

In addition to categorizing participants as described above, we also identified participants with multiple types of supplemental insurance coverages for sensitivity analyses.

eMethods 4. Covariates

Covariates of interest included demographic (age, gender, race/ethnicity), socioeconomic (income relative to the federal poverty line for each survey year, metropolitan vs rural area of residence, living arrangement/house composition (e.g. alone, spouse, family, other), and presence of chronic health conditions (including stroke, chronic obstructive pulmonary disease, depression, diabetes, chronic heart failure, hip fracture, hypertension, arthritis, ischemic heart

© 2024 Bessen S et al. *JAMA Health Forum.*

disease, self-reported hearing loss). Participant age was derived from Medicare enrollment data. Data on gender, race and ethnicity, self-reported health, education, living arrangement, income, number of ADL difficulties, comorbid chronic conditions, and presence of functional hearing loss was derived from self-reported Medicare Current Beneficiary Survey data. Metropolitan versus rural area of residence was derived from the zip code of the participant's mailing address and US Census Bureau statistical data.

eReference

1. Ochieng N, Clerveau G, Cubanski J, Neuman T. *A Snapshot of Sources of Coverage Among Medicare Beneficiaries*. Kaiser Family Foundation https://www.kff.org/medicare/issue-brief/a-snapshot-of-sources-of-coverage-among-medicare-beneficiaries/

© 2024 Bessen S et al. *JAMA Health Forum.*
