# Supplementary material for: Use of Hearing Services in Traditional Medicare and Medicare Advantage
Source: JAMA Health Forum. 2024 Oct 25;5(10):e243619. doi: 10.1001/jamahealthforum.2024.3619 (PMC11581654; doi:10.1001/jamahealthforum.2024.3619)
Supplement: Supplement 2. — Data Sharing Statement [file jamahealthforum-e243619-s002.pdf]

Data Sharing Statement

Bessen. Use of Hearing Services in Traditional Medicare and Medicare Advantage. *JAMA Health Forum*. Published October 25, 2024. doi:10.1001/jamahealthforum.2024.3619

Data
Data available: Yes
Data types: Data (not involving human participants)
How to access data: The data that support the findings of this study are available from the corresponding author, SYB (sbessen2@jh.edu), upon reasonable request.
When available: With publication

Supporting Documents
Document types: None

Additional Information
Who can access the data: Researchers whose proposed use of the data has been approved
Types of analyses: For any reasonable purpose following review of request by authors
Mechanisms of data availability: After approval of a proposal or with investigator support
